# A rare case of Aerococcus urinae native valve endocarditis

**DOI:** 10.1099/acmi.0.000863.v4

**Published:** 2025-03-27

**Authors:** Sofie Goes, Kim Callebaut, Denis Pierard, Ingrid Wybo, Deborah De Geyter, Astrid Muyldermans, Jolien Geers, Laura Kerselaers, Thomas Demuyser

**Affiliations:** 1 Department of Microbiology and Infection Control, Vrije Universiteit Brussel (VUB), Universitair Ziekenhuis Brussel (UZ Brussel), Laarbeeklaan 101, 1090 Brussels, Belgium; 2 Department of Cardiology, CHVZ (Centrum voor Hart en Vaatziekten), Vrije Universiteit Brussel (VUB), Universitair Ziekenhuis Brussel (UZ Brussel), Laarbeeklaan 101, 1090 Brussels, Belgium

**Keywords:** *Aerococcus urinae*, endocarditis, native valve

## Abstract

**Background.***Aerococcus urinae* was initially considered a commensal of the urinary tract, but there is now increasing evidence for its involvement in urinary tract and systemic infections. *A*. urinae endocarditis has a non-negligible mortality rate and occurs mainly in patients with underlying conditions or the presence of extraneous material.

**Case presentation.** This report handles the case of a 65-year-old male with cardiac antecedents, who was admitted to the cardiology department after a syncope of unknown origin and diagnosed with severe mixed aortic valve disease and mitral valve sclerosis through the means of transoesophageal echocardiography (TEE). During hospitalization, the patient progressively deteriorated with the development of shortness of breath and an inflammatory syndrome. Both the urine and blood cultures showed growth of *A. urinae*. Treatment with piperacillin/tazobactam was started empirically. Repeated TEE showed evidence of endocarditis with vegetation and perforation of the mitral valve that required an emergency surgery with mitral valve repair. After surgery, gentamicin and penicillin G were administered for 48 h, followed by combined ceftriaxone/penicillin G treatment for 6 weeks. At first, flucloxacillin was also associated as the culture of the valve was negative. Finally, the 16S rRNA gene PCR on the valve tissue confirmed the *A. urinae* endocarditis.

**Conclusion.***A. urinae* is an underestimated cause of serious infections such as endocarditis. Urinary tract infections mainly in older men can be an entry point for this type of invasive infection.

## Data summary

Only confidential data are located at the University Hospital Brussels, Brussels, Belgium. All other data required for the review of this case report are presented in the manuscript.

## Introduction

*Aerococcus urinae* is a Gram-positive coccus that was previously believed to be a contaminant in urinary culture, without clinical significance. Today, it is gaining recognition as a potential causative agent of urinary tract infections to invasive infections such as endocarditis [1]. Among all urinary tract infections, 0.2–0.8% are attributed to *A. urinae;* however, its association with endocarditis has been rarely documented. In this case report, we present an *A. urinae* endocarditis in an elderly individual.

## Case report

A 65-year-old man was admitted to a tertiary care centre in Brussels (Belgium), following syncope under unclear circumstances. The man lived independently and had an important psychiatric background, including former ethylic, bipolar disorder and suicidal tendencies. Since the patient appeared awake but was unresponsive after the syncope, communication was difficult. Three weeks prior to hospitalization, the man had already fallen down the stairs and developed persistent weakness in his right leg.

**Fig. 1.**
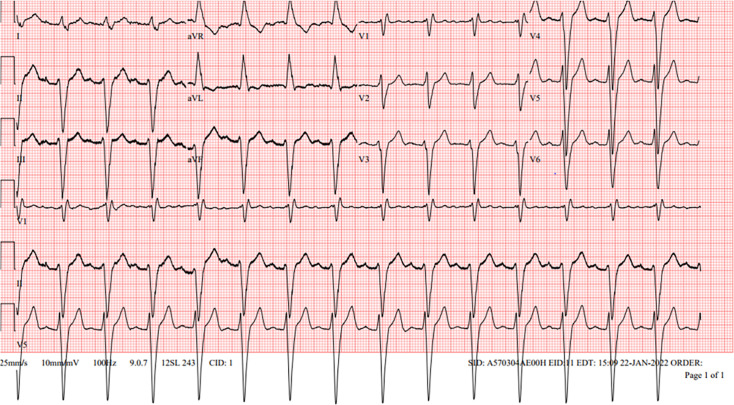
ECG of day 1 showing a sinus rhythm with a first-degree AV block and prolonged QRS interval (182 ms). ECG, electrocardiogram.

The patient suffered from diabetes mellitus type 2, well controlled at admission with an HbA1c value of 6.6%, and had multiple cardiovascular co-morbidities: arterial hypertension, hypercholesterolaemia, ex-smoking and ischaemic cardiomyopathy treated with multiple percutaneous coronary interventions. Upon arrival, he was hypotensive with a blood pressure of 96/59 mmHg and tachycardic, with a heart rate of 116 beats per minute. An urgent electrocardiogram was performed, revealing a sinus rhythm with first-degree atrioventricular block (Fig. 1) and unspecified prolonged QRS (Q-, R-and S-wave) interval. A prolonged QT (Q-and T-wave) interval could not be excluded; therefore, clotiapine was halted, valproic acid levels were monitored and levomepromazine dose was reduced.

During hospitalization, the patient developed several episodes of alternating bradycardia and tachycardia, leading to the diagnosis of sick sinus syndrome. Through the means of transoesophageal echocardiography (TEE), the patient was diagnosed with severe mixed aortic valve disease with severe aortic insufficiency and moderate to severe aortic stenosis with underlying bicuspid aortic valve and mitral valve sclerosis. Invasive coronary angiography revealed a stent thrombosis of the right coronary artery. Therefore, semi-urgent surgery was indicated with aortic valve replacement, coronary artery bypass graft (CABG) and pacemaker implantation. Further assessment of the patient included a brain computed tomography (CT) scan showing an old ischaemic lesion left in the basal frontal lobe. There were no arguments for epileptic insult and chest CT imaging ruled out pulmonary emboli.

Laboratory results at admission depicted a normal platelet count, haemoglobin, leucocyte count with a slightly elevated neutrophil count, a decreased red blood cell count, elevated d-dimer and cardiac troponin T being also elevated, as illustrated in Table 1. Toxicology analysis of ethanol was negative on admission.

**Table 1.** Laboratory results at admission

Laboratory test	Result	Reference range
Platelet count	174×10^3^ mm^−3^	163–347×10^3^ mm^−3^
Haemoglobin	13.6 g dl^−1^	13.50–17.00 g dl^−1^
Leucocyte count	7.90×10^3^ mm^−3^	4.30–9.64×10^3^ mm^−3^
Neutrophil count	6.53×10^3^ mm^−3^	1.93–5.87×10^3^ mm^−3^
Red blood cell count	4.00×10^6^ mm^−3^	4.40–5.80×10^6^ mm^−3^
d-Dimers	157×10^1^ g ml^−1^	<500 ng ml^−1^
Cardiac troponin T	0.0520 µg l^−1^	<0.005 µg l^−1^

On day 16, additionally, blood and urine cultures were collected as the patient developed an inflammatory syndrome and was subfebrile without a known cause.

From the urinary samples, obtained on day 1 and day 16, Gram-positive cocci were cultured. *A. urinae* was identified using the Bruker Biotyper MALDI-TOF (Bruker Daltonics, Billerica, MA, USA).

**Fig. 2.**
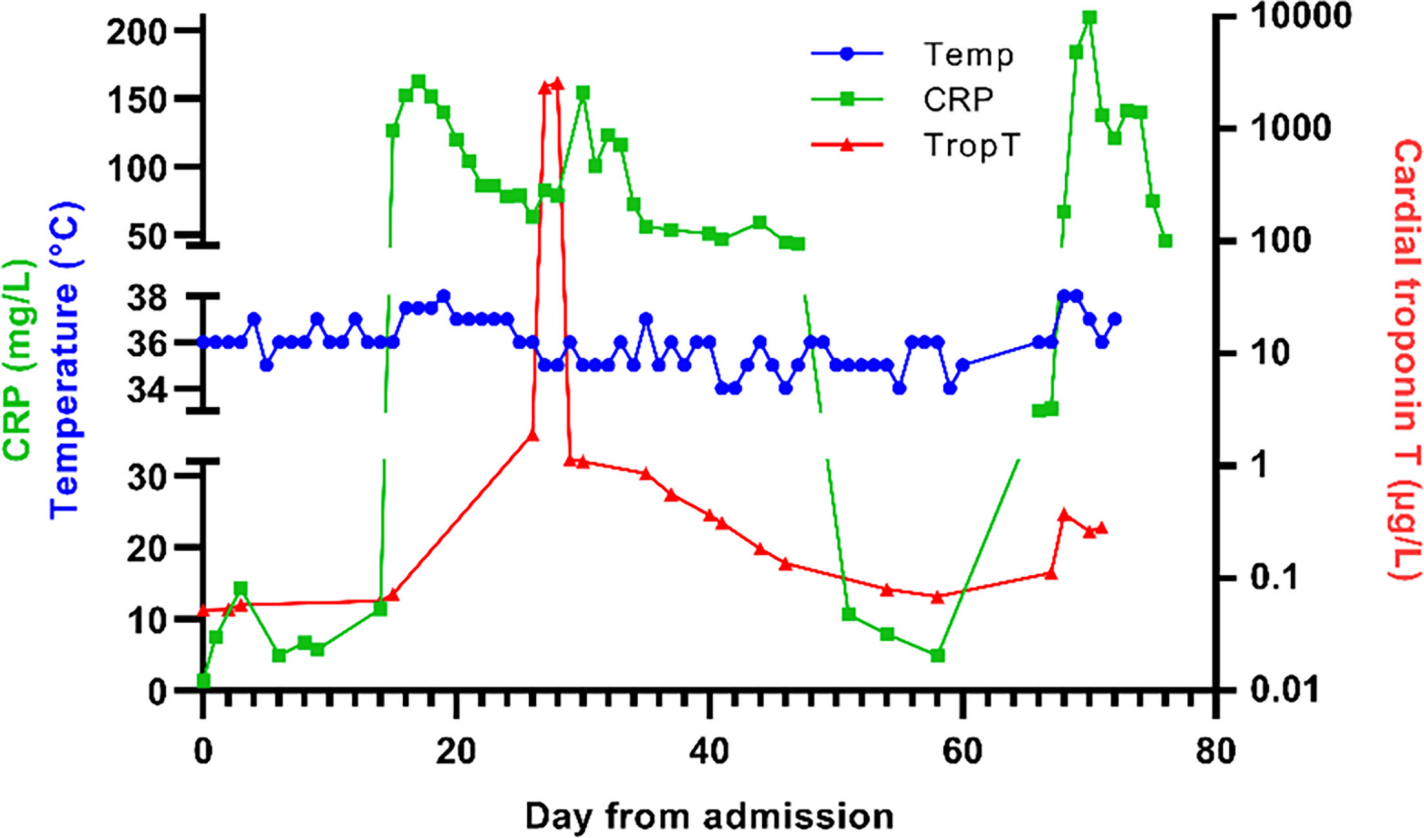
Changes in inflammatory marker (CRP), myocardial injury (Troponin T) and body temperature during hospitalization. Cut-off value for CRP and Troponin T is <5 mg l^−1^ and <0.005 µg l^−1^, respectively.

The day before the scheduled surgery (day 17 in hospital), the patient developed progressive chest pain and was platypnoeic. Because of increasing C-reactive protein (CRP) (Fig. 2), surgery was postponed. Following a suspicion of urosepsis and a subfebrile state, piperacillin/tazobactam (3×4/0.5 g) was started empirically on day 17, considering the urine culture demonstrated susceptibility to penicillin using the disc diffusion method for antimicrobial susceptibility testing. In his blood cultures, *A. urinae* was also isolated and penicillin sensitivity was observed. However, no evidence of perinephric abscess was found on the abdominal ultrasound. In addition, the peripheral intravenous catheter tip was sent to the lab, yet its culture remained negative. Three days later, the treatment was switched from piperacillin/tazobactam to amoxicillin per os (3×1 g) based on the susceptibility testing of the blood culture isolate.

**Fig. 3.**
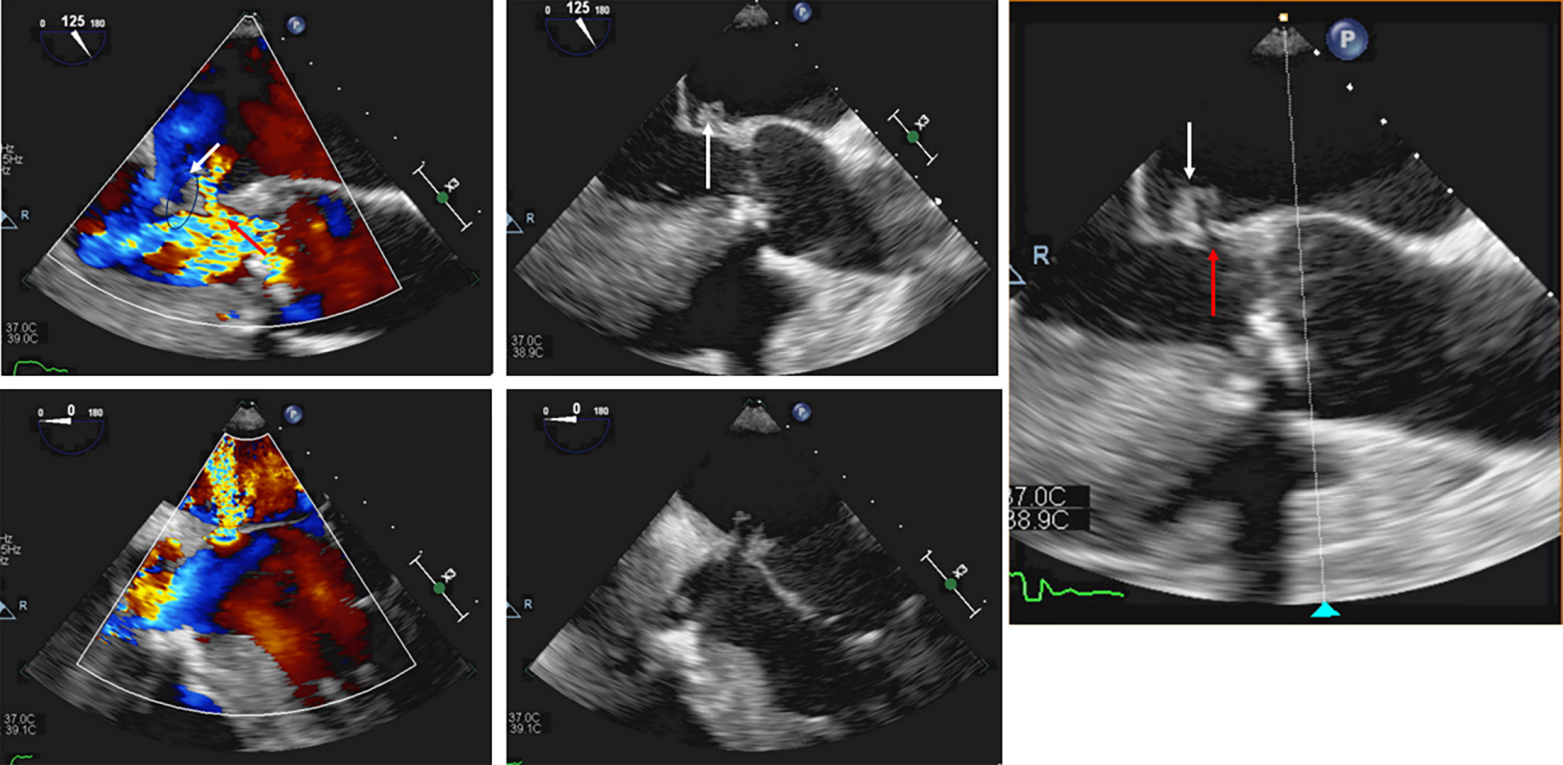
Images of the TEE examination performed in the patient showing vegetation (white arrows) and perforation (red arrows) of the mitral valve (a) and (d) are colour Doppler echocardiographic images clearly demonstrating the jet lesion through the perforation.

On day 26, the TEE showed evidence of endocarditis with mitral valve vegetation and perforation with a jet lesion (Fig. 3). Finally, on day 27, the surgery was carried out with repair and annuloplasty of the mitral valve, replacement of the aortic valve by a bioprosthesis, CABG and placement of a cardiac resynchronization therapy defibrillator. Gentamicin (3 mg kg^–1^) was administered intravenously (IV) combined with penicillin G IV (6×4 million IU) for 48 h post-operative. Due to impaired renal function (creatinine increased and estimated glomerular filtration rate decreased), gentamicin was replaced by ceftriaxone (2×2 g). Awaiting valve tissue cultures, collected during surgery, flucloxacillin IV (6×2 g) was associated to cover a potential methicillin-sensitive *Staphylococcus aureus-*associated endocarditis.

**Fig. 4.**
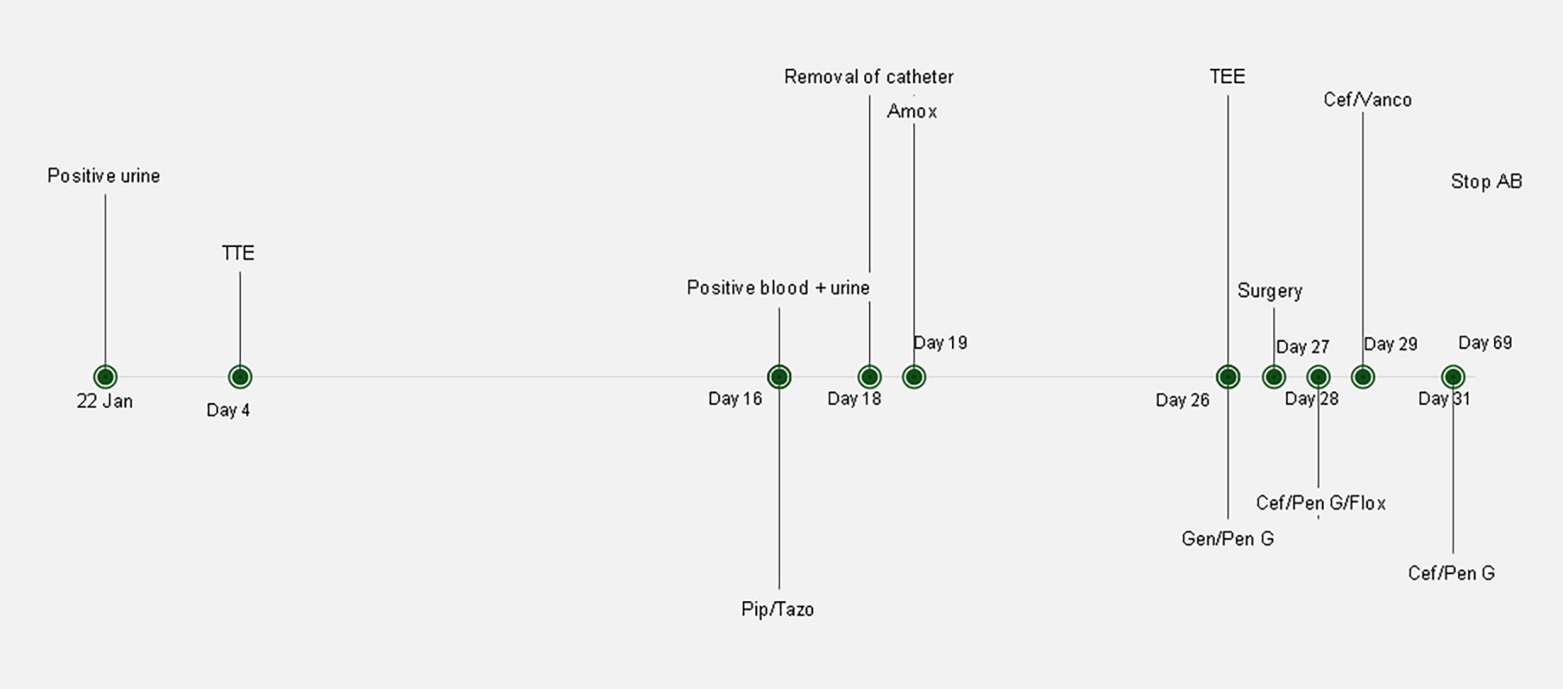
Timeline of the patient’s hospitalisation. AB, antibiotics; Amox, amoxicillin; Cef, ceftriaxone; Flox, flucloxacillin; Gen/Pen G, gentamicin/penicillin G.

However, cultures of the valve surgery remained negative. PCR (GeneAmp 9700 and ABI 3730xl, Thermo Fisher Scientific) of the valve targeting the 16S rRNA gene revealed *A. urinae* after which flucloxacillin was discontinued. Penicillin G and ceftriaxone were maintained for 6 weeks (Fig. 4) starting from the valve replacement, to adequately treat the endocarditis.

After 61 days in the hospital, the patient was discharged; however, 6 days later, he was readmitted to the intensive care unit. He suffered a massive cardiogenic shock due to mitral valve dislodgement but recovered after surgical reattachment.

## Discussion and conclusion

*A. urinae* can be considered part of the normal urinary tract flora, but in certain cases, it results in symptomatic urinary tract infections. This is particularly the case in men of advanced age and in patients who already have pre-existing underlying pathologies that increase the risk for urinary tract infections. These risks may include anything from prostatic hypertrophy and bladder cancer to the presence of catheters [2]. Once *A. urinae* is translocated into the systemic circulation, it can induce serious infections such as osteomyelitis, septic arthritis, spondylodiscitis, meningitis and endocarditis [3].

In the majority of native valve infective endocarditis (IE) cases, Gram-positive cocci are the causative agent, with *S. aureus* representing up to one-third of cases [4]. This is in contrast with the incidence of positive blood cultures with *A. urinae,* which is estimated at 3/1,000,000 per year [3,5]. This is probably an underestimation due to the limited description of endocarditis caused by *A. urinae* in the literature and misidentifications before the development of more accurate methods.

*A. urinae* was previously mistaken occasionally for *Streptococcus spp*. due to the appearance of alpha haemolysis on blood agar or for *Staphylococcus spp*. because of the similar morphology shown under the microscope. However, through the development of new diagnostic techniques, such as the MALDI-TOF and molecular techniques, pathogens can be identified with more accuracy [6].

As with *S. aureus*, *A. urinae* easily settles into the endocardium in the presence of valve disease, disturbed blood flow or the presence of foreign material in the heart [3]. The virulence strategies of *A. urinae* resemble those of *Streptococci*, like the ability to form biofilms and induce platelet aggregations [7,8].

Endocarditis can be diagnosed based on echocardiography findings, symptoms such as fever, the presence of septic emboli and positive blood cultures [9,11].

In this patient, the blood and urine cultures were positive, but the culture of the valve itself remained negative. Low sensitivity (25.4%) and specificity (71.6%) of the culture of heart valves in endocarditis patients have already been described [12].

Negative culture results of heart valves are often due to previous antibiotic treatment or by fastidious organisms, for example, aerococci that demand certain components to grow sufficiently. In this case, the patient had been previously administered adequate antibiotic treatment possibly explaining the valve’s negative culture. Even though the amount of organisms remaining was limited, the DNA required to identify the germ by 16S rRNA PCR was still sufficient [13,14].

The sensitivity and specificity of 16S rRNA gene PCR in culture-negative endocarditis vary from 41–61% and 100%, respectively. The latter was higher than conventional culture (7.8–13% sensitivity and 98–100% specificity) [15,16]. The ESC guidelines and the 2023 DUKE ISCVID criteria also state the addition of molecular tests, such as fluorescence *in situ* hybridization, which in combination with 16S rRNA gene PCR could enhance detection [17,18].

The susceptibility of *A. urinae* overall varies slightly. It is sensitive *in vitro* to penicillin and is often resistant to sulphonamides and fluoroquinolones [19]. *In vitro*, a synergy was also observed between penicillin and gentamicin [14]. An unusual antibiotic association was used in this case of penicillin with ceftriaxone. For *Enterococcus faecalis-associated IE*, ampicillin combined with ceftriaxone is an alternative for aminoglycoside-resistant strains, yet there is no evidence to support this being equally effective in *A. urinae* IE [17].

As discussed earlier, 16S rRNA gene PCR can detect bacterial DNA even after complete antibiotic treatment. However, the detection of bacterial DNA may not indicate the existence of endocarditis in progress. Note that there is no golden diagnostic standard for endocarditis. It is rather an assessment of the combination of symptoms, imaging, laboratory findings and cultures to evaluate [17,18]. Molecular tests should always be interpreted in context and certainly not used alone.

Infectious endocarditis with *A. urinae* overall has a good prognosis relative to IE caused by other bacteria [20,21]. The number of *A. urinae* endocarditis cases has been underestimated, but the incidence is increasing with progress in the development of diagnostic methods. Because of the limited literature on severe infections with *A. urinae*, there are currently no official guidelines regarding diagnosis and treatment. Endocarditis should be considered in the case of aerococcal septicemia, especially in the case of older male patients who suffer from urinary tract infections.

## Abbreviations

AVR: aortic valve replacement
BAV: bicuspid aortic valve
CABG: coronary artery bypass graft
CRP: C-reactive protein
CRT-D: cardiac resynchronization therapy with a defibrillator
CT: computed tomography
ECG: electrocardiogram
IV: intravenously
MALDI-TOF: matrix assisted laser desorption Ionization time of flight
PCR: polymerase chain reaction
QRS: Q-, R-and S-wave
RCA: right coronary artery
TEE: transoesophageal echocardiography
